# Digitally managing depression: A fully remote randomised attention-placebo controlled trial

**DOI:** 10.1177/20552076241260409

**Published:** 2024-06-07

**Authors:** Aaron Kandola, Kyra Edwards, Marie AE Muller, Bettina Dührkoop, Bettina Hein, Joris Straatman, Joseph F Hayes

**Affiliations:** 1 MRC Unit of Lifelong Health and Aging, University College London - UCL, UK; 2 juli Health, Hull, MA, USA; 3 Division of Psychiatry, University College London - UCL, UK; 4 Camden and Islington NHS Foundation Trust, London, UK

**Keywords:** Digital health, behaviour change, self-monitoring, depression, digital clinical trials, Apps, motivation

## Abstract

**Background:**

Depression is a common and disabling condition. Digital apps may augment or facilitate care, particularly in under-served populations. We tested the efficacy of juli, a digital self-management app for depression in a fully remote randomised controlled trial.

**Methods:**

A pragmatic randomised controlled trial that included participants aged > 18 who self-identified as having depression and scored > 5 on the Patient Health Questionnaire-8. Participants were randomly assigned (1:1) to receive juli for 8 weeks or a limited attention-placebo control app. Our primary outcome was the difference in Patient Health Questionnaire-8 scores at 8 weeks. Secondary outcomes were remission, minimal clinically important difference, worsening of depression, and health-related quality of life. Analyses were per-protocol (primary), and modified and full intention-to-treat (secondary). The trial was registered at ISRCTN (ISRCTN12329547).

**Results:**

Between May 2021 and January 2023, we randomised 908 participants. 662 completed the week 2 outcome assessment and were included in the modified intention-to-treat analysis, and 456 completed the week 8 outcome assessments (per-protocol). In the per-protocol analysis, the juli group had a greater reduction in Patient Health Questionnaire-8 score (10.78, standard deviation 6.26) than the control group (11.88, standard deviation 5.73) by week 8 (baseline adjusted β-coefficient −0.94, 95% CI: −1.87 to −0.22, *p* = 0.045). Achieving remission and a minimal clinically important difference was more likely in the juli group at 8 weeks (adjusted odds ratios 2.22, 95% CI: 1.45–3.39, *p* < 0.001 and 1.56, 95% CI: 1.08–2.27, *p* = 0.018, respectively). There were no between-group differences in health-related quality of life or worsening of depression. Modified and full intention-to-treat analyses found similar results, but the primary outcome was non-significant.

**Conclusion:**

The use of juli for 8 weeks resulted in a small reduction in symptoms of depression compared with an attention-placebo control. The juli app is a digital self-management tool that could increase the accessibility of evidence-based depression treatments.

## Introduction

Depression is a major contributor to the global burden of disease. Each year, millions of people are diagnosed with depression. It can frequently become chronic and recurrent and is likely to be the leading cause of disability in high-income countries by 2030.^1^ The mainstay of treatment for depression includes psychological therapy and antidepressant medication. Barriers to accessing care include high costs, low availability, long waiting lists and stigma.^2^

Self-management support has become a central tenet of chronic illness management.^3^ Supported self-management means increasing the knowledge, skills and confidence a person has in managing their own health. There is increasing evidence for this approach in augmenting the treatment and management of depression.^4^ Self-management in depression includes health awareness, education, medication adherence support, behavioural activation and other approaches from cognitive behavioural therapy (CBT).^5^ Digital self-management interventions may remove some of the barriers present in accessing depression care by offering scalable solutions that are convenient and timely.^6^ However, there has been little assessment of the efficacy of digital self-management support for people with depression.

Recent meta-analyses of randomised controlled trials (RCTs) of a smartphone application (app)-based psychological interventions for depression symptoms find a small to moderate reduction in symptoms compared to a placebo, such as waiting list control.^7,8^ However, subgroup analyses found no difference in depressive symptoms in studies with active control groups, which is corroborated by the findings of other systematic reviews.^9^ The majority of the apps included offer time-limited digital courses of CBT, rather than ongoing self-management support.^8^ Many of the apps included in the reviews are no longer (or have never been) commercially available, highlighting that translation from research to clinical impact is difficult in this space. Other challenges include low levels of retention and engagement with digital health apps in trials and real-world use.^10^

The digital health app juli, aims to support people with depression via evidence-based approaches, including mood tracking, medication reminders, positive affect journaling, data visualisation of sleep, activity, exercise, heart rate variability and behavioural activation recommendations about how to improve these parameters.^11^ Digital biomarkers included in juli have been found to aid tracking of depression severity and treatment response.^12–14^ As such, juli combines many of the elements that have been found to be effective in research-grade apps for depression.^7,8^ However, there has been less evaluation of consumer-grade apps in real-life practice. This is important as many popular health apps have not been scrutinised in the way new interventions traditionally would be.

We hypothesised that individuals randomised to juli would have a greater reduction in depression symptoms at 8 weeks than those in an attention placebo control group. Our RCT was fully remote, increasing cost-effectiveness, time efficiency and reach.

## Methods

### Study design and participants

We conducted a fully remote pragmatic placebo control randomised controlled trial of people with depression from anywhere in the world. Individuals were eligible for inclusion if they were aged 18 to 65, were English speaking, had access to an iPhone, and self-identified as having depression, with a score of 5 or more on the Patient Health Questionnaire 8-item version (PHQ-8) at baseline. A score of more than 4 is consistent with a current diagnosis of mild depression. There was no upper limit to the PHQ-8 score, so we could recruit participants with a range of depression severities. We recruited participants via online adverts, social media posts and self-help groups for depression. Recruitment was from May 2021 until January 2023. All participants provided written informed consent via a consent form within the app. Ethical approval was from the University College London Ethics Committee (ID number 19413/001). The trial was registered on the ISRCTN registry (https://doi.org/10.1186/ISRCTN12329547).

### Randomisation and masking

We randomly assigned participants (1:1) to a full version of juli or an attention-placebo control version. Block randomisation was conducted within the app using automated code, with random block sizes between 4 and 8. The researchers and independent statisticians were masked to treatment allocation until the completion of the analysis, with randomisation being completed remotely and this information only being held by a data engineer independent from the trial.

### Procedures – The juli app

**Figure 1.**
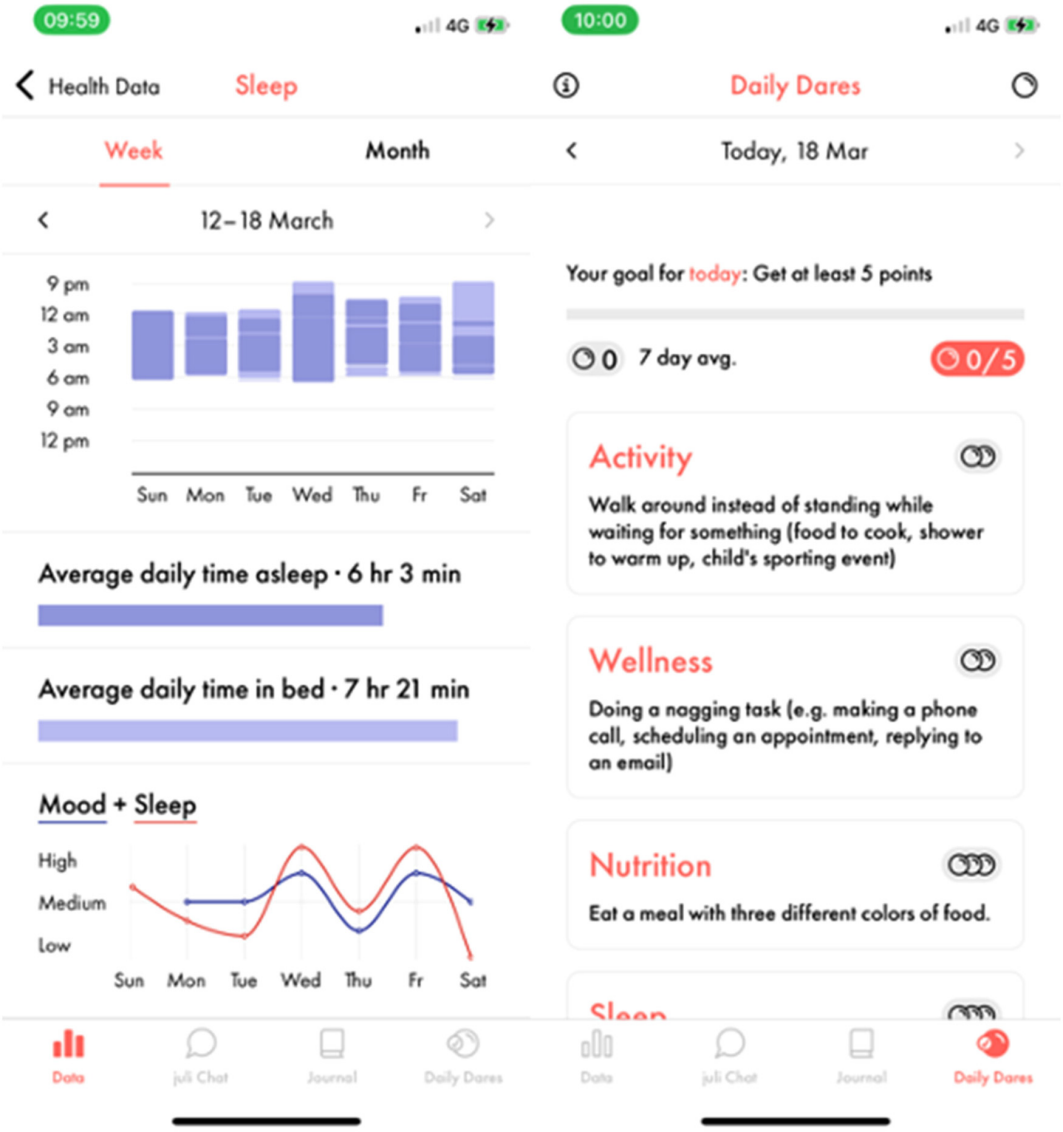
Example juli screenshots.

Participants were either allocated to the full version of the juli app or an attention placebo control app group. If allocated to the full version of the app, participants were prompted to open the app each day via an automated alert at the time of their choosing. They were asked to rate how they were feeling on a scale using five emoji faces and a circumplex model with mood on the *x*-axis and energy on the *y*-axis.^15^ Individuals were also able to track things that they considered as important contributors to their mood.^16^ The app passively gathered information via smartphone and smartwatch sensors on sleep, activity, workouts, menstrual cycle, and heart rate variability on a daily basis and presented this data to the participant, showing associations with mood.^17^ The app then provided recommendations about these parameters to guide healthy behaviours via behavioural activation.^18^ The app includes a medication reminder function that can be set by the participants to improve medication adherence.^19^ Participants were also encouraged to engage in positive affect journaling via the app (Figure 1).^20^ The juli app was designed by a psychiatrist and experts in gamification.^21^ It is grounded in evidence-based treatments. Participants were guided towards all elements of the app, but did not have to engage with elements they did not like. If allocated to the control arm, participants were prompted to open the app each day via an automated alert and to rate how they were feeling on a scale using five emoji faces. Control participants did not receive any further intervention. Control participants were invited to use the full app after the end of the trial.

Baseline assessments and follow-up assessments at 2, 4, 6 and 8 weeks were all completed within the app. At baseline, participants were asked their age, gender, duration of depression, whether this was diagnosed by a physician, if they took an antidepressant and if they remained in contact with a physician for reviews of their mental health.

### Questionnaires used

Participants completed the PHQ-8 for depression symptoms and the 12-Item Short Form Health Survey (SF-12) for health-related quality of life at baseline. The PHQ is a widely used self-completed depression scale that is recommended by the Common Measures in Mental Health Science Governance Board as one of a core list of research questionnaires that should be used by funded researchers.^22^ The PHQ-9 closely matches the DSM-IV criteria for a major depressive episode and may be more sensitive to change than other measures of depression, such as the Hamilton Rating Scale for Depression and the Beck Depression Inventory.^23^ The PHQ-8 excludes the question about suicidality, which is preferred in studies where patient contact is remote, such as via digital technologies or telephone. Research indicates that the deletion of this question has little effect on the scale's psychometric properties because this question is the least frequently endorsed item on the PHQ-9. Subsequently, the PHQ-8 has identical scoring thresholds for depression severity, with higher scores representing more severe depression.^24,25^ The sensitivity and specificity of a PHQ-8 score ≥ 10 for major depressive disorder is 100% and 95%, respectively.^24^ The SF-12 is a self-reported health status measure.^26^ Possible scores range from 0 to 100, with higher scores indicating better quality of life.

### Primary outcome

The primary outcome was the total score on the PHQ-8 at 8 weeks. Despite the often chronic nature of depression assessment of treatment efficacy has regularly been at 8 weeks as 4–8 weeks is often the earliest a treatment response can be observed.^27^

### Pre-specified secondary outcomes

The secondary outcomes were (1) PHQ-8 score as a continuous outcome at 2, 4, 6 and 8 weeks in a repeated measures analysis, (2) PHQ-8 score as a binary outcome where remission is a score of <10 at 8 weeks,^28^ (3) remission at 2, 4, 6 and 8 weeks in a repeated measures analysis, (4) Difference in SF-12 physical and mental component scores at 8 weeks and, (5) SF-12 physical and mental component scores at 4 and 8 weeks in a repeated measures analysis.

### Post-hoc secondary outcomes

We added post-hoc outcomes that included (1) achieving a minimal clinically important difference (MCID) at 8 weeks defined by the effective dose 50 method, which accounts for baseline severity and is the smallest difference in PHQ-8 scores that are of perceived benefit,^29^ (2) a worsening of depression, defined as a >20% increase in PHQ-8 from baseline.^30^ We performed a sensitivity analysis for the endpoint PHQ-8 score as a binary outcome where remission is a score of <10 at 8 weeks, including only individuals who had a PHQ-8 score of > 9 at baseline (as some individuals may not have been able to achieve this outcome, because of their low baseline score).

### Sample size

At the time of planning this RCT, the best estimate of an MCID as measured by the PHQ-8 was between 11% and 14%, with a standard deviation of 0.32–0.38.^31,32^ To observe an 11% mean difference between intervention and control arms with 80% power at the two-sided 5% significance level required a total sample size of 189 per arm for the per-protocol analysis. Allowing for 26% attrition,^33^ we aimed to randomise 238 participants per arm. Power calculations were carried out using Stata.

### Statistical analyses

Our analysis plan was pre-printed (https://discovery.ucl.ac.uk/id/eprint/10129350/) and included in the ISRCTN registry. We followed the Consolidated Standards of Reporting Trials (CONSORT) guidelines in reporting and analysing our data.^25^ Primary and secondary outcomes were described in the published protocol and on the ISRCTN registry before the study started.

The primary outcome was the difference in change in total PHQ-8 score at 8 weeks between control and intervention groups in a per-protocol analysis. This was estimated with a linear regression model adjusted for baseline PHQ-8, as a continuous variable. We calculated the odds ratio of remission at 8 weeks (PHQ-8 < 10), achieving MCID and worsening of depression, adjusting for baseline severity using logistic regression. Repeat measures analyses were completed using linear or logistic mixed effect models adjusting for baseline severity with an intercept at the participant level.

**Figure 2.**
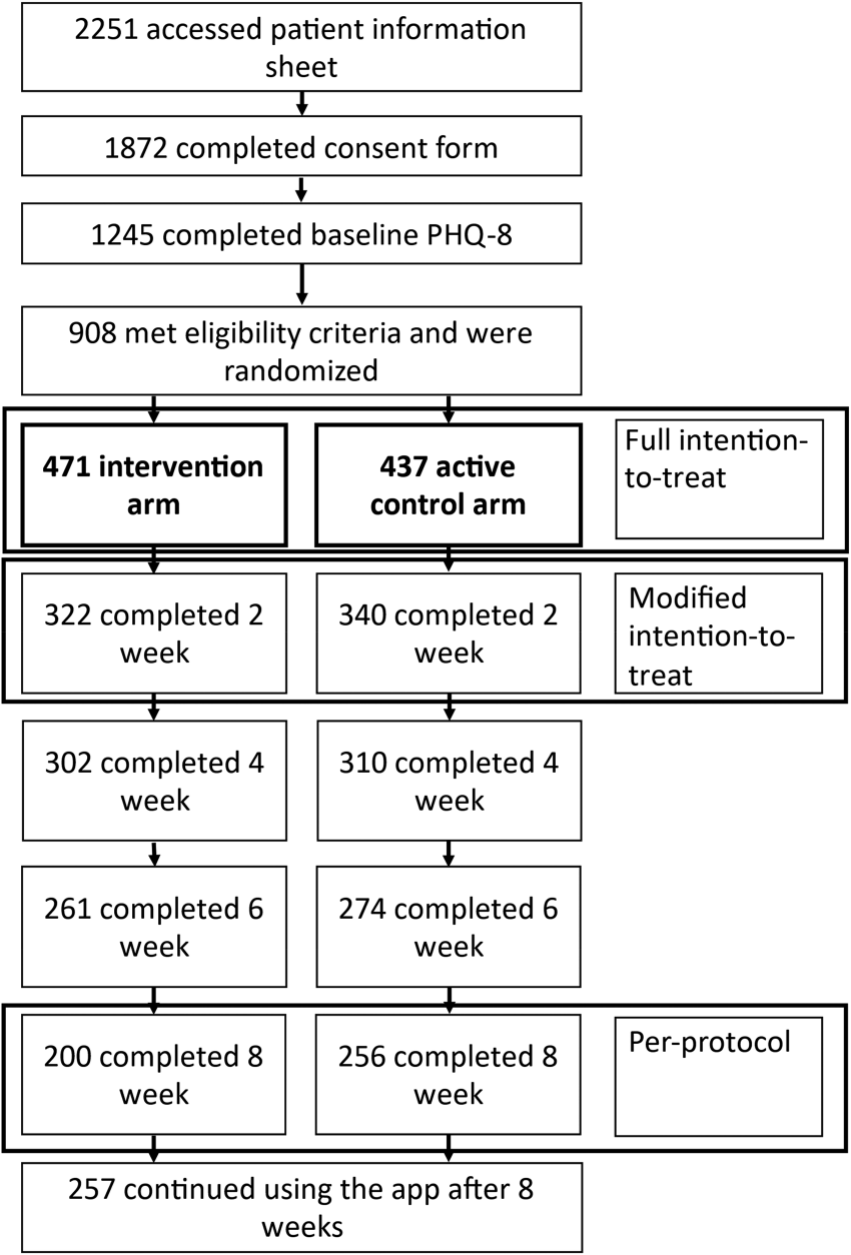
Consolidated Standards of Reporting Trials (CONSORT) diagram.

We also examined all outcomes in a modified intention-to-treat analysis including all randomised participants with a complete baseline and week 2 PHQ-8, therefore dropping individuals who were randomised but never used the app, and a full intention-to-treat analysis including all randomised participants (Figure 2). Missing outcome data were imputed by (i) multiple imputation and (ii) last observation carried forward.^34^ Multiple imputation was completed via predictive mean matching with five nearest neighbours and 50 iterations. Predictive mean matching has been shown to be more robust to model misspecification than fully parametric imputation methods and only plausible values are imputed.^35^

All analyses were completed by independent statisticians using Stata and R, who have no financial conflict of interest with the company providing the juli app.

## Results

### Per-protocol analysis

We recruited 456 individuals who were retained in the trial for 8 weeks and formed the basis of our primary per-protocol analysis (Figure 2). The majority of participants were female and had experienced depression for more than 5 years (Table 1). The majority were diagnosed by a physician and continued to be in regular or occasional contact with a doctor about their depression (Table 1). The mean PHQ-8 score at baseline was 16.16 (standard deviation (SD) 4.71). The mean number of times participants entered data in the app in the intervention arm was 4.45 per week (SD 1.77) and in the control arm was 4.84 (SD 1.59). There was no difference between groups in terms of app openings (*p* = 0.014). The mean app usage time was 17.80 min per week (SD 7.09) in the juli arm and 2.42 min per week (SD 0.79) in the control arm.

**Table 1.** Baseline characteristics.

	Intervention (n = 200)	Control (n = 256)	All (n = 456)
Age	34.39 (12.41)	32.65 (12.28)	33.41 (12.35)
Gender			
Female	154 (77.00)	202 (78.91)	356 (78.07)
Male	33 (16.50)	37 (14.45)	72 (15.69)
Transgender or non-binary	13 (6.50)	17 (6.64)	30 (6.58)
Depression duration			
< 1 month	1 (0.50)	1 (0.39)	2 (0.44)
1 to < 3 months	7 (3.50)	2 (0.78)	9 (1.97)
3 months to < 1 year	2 (1.00)	14 (5.47)	16 (3.51)
1 to < 2 years	11 (5.50)	15 (5.86)	26 (5.70)
2 to < 5 years	43 (21.50)	49 (19.14)	92 (20.18)
> 5 years	136 (68.20)	175 (68.36)	311 (68.20)
Physician contact			
Regular	76 (38.00)	112 (43.75)	188 (41.23)
Occasional	67 (33.50)	64 (25.00)	131 (28.73)
Not anymore	32 (16.00)	38 (14.84)	70 (15.35)
Never	25 (12.50)	42 (16.41)	67 (14.69)
Prescribed antidepressant			
Yes	161 (80.50)	192 (75.00)	353 (77.41
No	39 (19.50)	64 (25.00)	103 (22.59)
Diagnosed by a physician			
Yes	179 (88.61)	219 (85.55)	397 (87.06)
No	22 (11.00)	37 (14.45)	59 (12.94)
PHQ-8 total score^a^	16.09 (4.94)	16.30 (4.67)	16.21 (4.78)
SF-12 physical health subscale^b^	46.61 (9.29)	45.05 (9.81)	45.74 (9.61)
SF-12 mental health subscale^c^	22.15 (7.87)	22.09 (7.70)	22.12 (7.79)

Data are *n* (%) or mean (SD).

^a^ Patient Health Questionnaire, 8-item version (possible range 0–24).

^b^ Short-Form Health Survey12 physical health subscale (possible range 0–100).

^c^ Short-Form Health Survey-12 mental health subscale (possible range 0–100). Data used in the per-protocol analysis of individuals completing week 8 PHQ-8.

**Figure 3.**
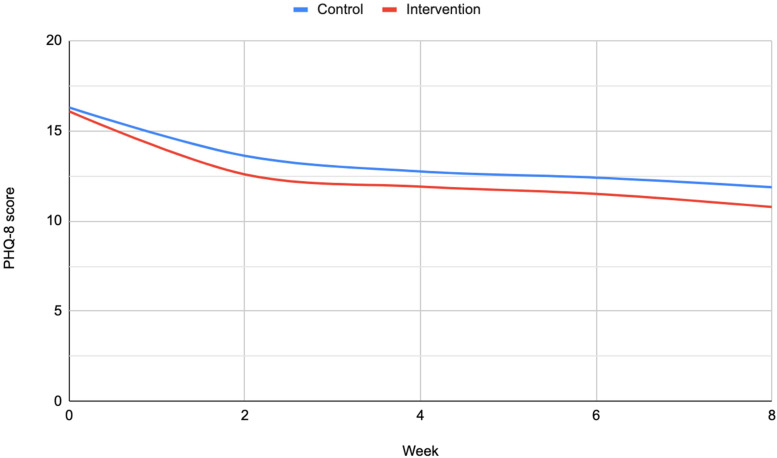
Mean Patient Health Questionnaire 8-item version (PHQ-8) score from baseline to week eight.

At 8 weeks, participants in the intervention group had a mean PHQ-8 score of 10.78 (SD 6.26) and participants in the control group had a mean of 11.88 (SD 5.73) (Figure 3). After accounting for baseline PHQ-8 score the intervention group had a greater reduction in depression symptom scores at 8 weeks (−0.94, 95% confidence interval (CI): −1.87 to −0.22, *p* = 0.045). The odds of being in remission by week 8 were higher in the intervention group after accounting for baseline depression severity (adjusted odds ratio 2.22, 95% CI: 1.45–3.39, *p* < 0.001). Participants in the intervention group were more likely to experience MCID (adjusted odds ratio 1.56, 95% CI 1.08–2.27, *p* = 0.018) than those in the control group. Repeat measures analyses of these outcomes at 2, 4, 6 and 8 weeks suggest that this effect was maintained over time (Supplemental Table 1). We found no effect of the intervention on SF-12 mental or physical component scores. The odds of experiencing worsening symptoms were similar in the intervention and control groups (adjusted odds ratio 0.83, 95% CI: 0.38–1.81, *p* = 0.633).

### Modified intention-to-treat analysis

Modified intention-to-treat analysis included 322 participants in the intervention group and 340 controls who completed baseline and week 2 PHQ-8 (Supplemental Table 2). The baseline characteristics of participants in these groups were similar to the per-protocol analysis (Supplemental Tables 2 and 3). In the multiple imputation data set, there was no clear difference between intervention and control groups after accounting for baseline severity (β-coefficient −0.78, 95% CI: −1.61 to 0.04, *p* = 0.063) (Supplemental Table 1) at week 8. However, the repeated measures analysis of PHQ-8 scores found a positive effect of the intervention on depression symptoms (−0.65, 95% CI: −1.21 to −0.09, 0.022). The odds ratios for remission at 8 weeks and remission in repeated measures were similar to the per-protocol analysis, and suggested higher odds of remission in the intervention group (adjusted odds ratio 1.94, 95% CI: 1.32–2.83, *p* = 0.001). The odds ratios for MCID and worsening of symptoms were consistent with the per-protocol results. Results from the last observation carried forward data set were consistent with the multiple imputation findings (Supplemental Table 1).

### Full intention-to-treat analysis

In total, 908 participants were randomised. Of these, 246 (27%) did not use the app after completing baseline questionnaires (Figure 2). The baseline characteristics of this group were similar to the modified intention-to-treat and per-protocol groups (Supplemental Table 3). In both the multiple imputation data set and the last observation carried forward data set, the difference in PHQ-8 at 8 weeks was non-significant. Odds of remission at 8 weeks and remission in repeated measures were elevated in the intervention arm.

## Discussion

We found a small mean reduction in depression symptoms in participants using juli for 8 weeks compared to an attention-placebo control, consistent with approximately a one-point improvement in symptoms as measured by the PHQ-8. Despite this small difference, participants allocated to juli were more than twice as likely to be in remission by 8 weeks, and more likely to meet the threshold for MCID in depression symptoms, taking their baseline severity into account. Results were consistent in our full and modified intention-to-treat analyses, but our primary endpoint was non-significant.

The participants had a mean baseline PHQ-8 score consistent with a diagnosis of moderately severe depression, the majority had longstanding depression and were under the ongoing care of a physician and were taking antidepressants. This is important as our participants differ from those included in many digital depression intervention RCTs in terms of experiencing more severe and long-lasting depressive symptoms.^7^ This does not suggest that people were accessing juli because of an absence of traditional care. Individuals with no previous or ongoing healthcare may differentially benefit from digital technologies. Digital apps could be one solution to an overburdened healthcare system, particularly in groups or areas where accessing treatment for mental health conditions is challenging or stigmatising.^36^ However, some people may still be unwilling or unable to use apps for health, despite their relative ease of access. The majority of participants were female, which reflects established differences in sex-specific rates of depression and help-seeking for depression.^37^ Some of our participants (∼7%) identified as transgender or non-binary. This group is under-represented in research but has a higher risk of depression and other mental disorders.^38^ Our high uptake suggests that digital technologies may be a better way of engaging these populations.

The improvement in the attention-control group was of a similar magnitude to other placebo-controlled trials of depression interventions.^39^ In addition, even basic mood monitoring, as required by our control group, has been found to decrease depression symptoms.^40^ This may have reduced the difference observed between intervention and control groups, compared to an inactive control (such as a waiting list).

The small improvement in depression symptoms observed by participants randomised to juli and still using it at 8 weeks needs to be considered in the context of existing treatments for depression.

The effect in this RCT is consistent with a standardised mean difference (SMD) of 0.16. Network meta-analyses have found newer antidepressants have an SMD of 0.30 compared to placebo^41^ and a recent high-profile RCT of sertraline in a mixed-severity population, like ours, found no difference in the PHQ-9 between the sertraline-treated group and placebo at 6 weeks.^32^ Unguided self-help CBT has an SMD of 0.13 compared to treatment as usual.^42^ As such, juli's efficacy as an augmentation to regular care; providing timely self-help CBT and medication reminders to approve adherence is of similar magnitude to recommended depression interventions.

Findings from meta-analyses support the use of digital technologies for the treatment of depression.^7–9^ However, many of the interventions reviewed are not available to patients, often because they are not available commercially or via healthcare providers. juli uses a combination of evidence-based approaches to support symptom reduction in depression and is available globally in Apple and Android formats.

### Strengths and limitations

Our RCT has a number of strengths and limitations. We successfully recruited, screened, randomised, treated, and assessed a geographically dispersed sample of participants. We modified the juli app so that RCT participants could consent, be randomised and take the baseline assessment within the app. This facilitated global recruitment, at a low cost, in a pragmatic manner, with good external validity. However, this also meant that we lacked information on potentially important baseline characteristics, such as social determinants of health, as we did not want to overburden the participants. Additionally, despite the availability of juli on both Android and Apple platforms, we only recruited participants who had an iPhone for the RCT, and as such included participants may not be generalisable to the wider population with depression. Balance in the recorded baseline characteristics after randomisation supports the assumption that randomisation was successful. Additionally, we did not include a large battery of outcome measures, which may have shed further light on our findings. For example, improvements in anxiety symptoms have been found in RCTs of interventions for depression.^32^

Attrition was higher than we anticipated (49.78% from randomisation to week 8). The majority of the attrition occurred between randomisation and week 2, which is common in RCTs, including for depression apps.^33^ A recent meta-analysis of dropout rates found similar attrition after adjusting for publication bias.^33^ We overcame this by recruiting until we had sufficient numbers who had completed the week 8 outcome measures and examined differences in completers vs non-completers. However, this attrition threatens the validity of our results as both the full intention-to-treat analysis, from the point of randomisation and the modified intention-to-treat analysis required a large amount of data to be imputed. The majority of early attrition was from participants who never began to use the app. To reduce this, future RCTs of digital interventions may benefit from a run-in period, in which participants become familiarised with the app before randomisation.^43^ We did not collect information about other reasons for dropout. Ideally, we would like to examine if any treatment effects are maintained over a longer time period. The difference in app usage time between the juli and control arms suggests that participants may have been aware of which group they had been allocated to.

An even bigger problem than the high attrition in RCTs of digital apps for depression is their lack of real-world retention and engagement. The proportion of 30-day retention is < 10% across mental health apps.^10^ This highlights that engagement and the potential clinical benefit can be increased by methods employed by juli, which has higher retention. However, it is unclear which specific features increase engagement as we did not collect usage data beyond the frequency participants opened the app and passively entered data. For example, a 2021 meta-analysis found no benefit of gamification.^44^ More research is needed to understand which depression app features are integral to improving mental health symptoms and how to optimise these for maximum effectiveness.

We analysed participants using the app for 8 weeks in our primary analysis to focus on the effects of maintained use. Baseline characteristics were similar in the full intention-to-treat and modified intention-to-treat, suggesting little difference between completers and non-completers (Supplemental Table 3). Following the publication of our protocol and commencement of our RCT, newer research on the PHQ-8 MCID was published suggesting that the MCID varies by baseline depression severity.^29,45^ We used these newer methods to derive a post hoc MCID outcome.^29^ We used two imputation methods for the intention-to-treat analyses that make different assumptions.^34^ Results from both methods did not differ.

## Conclusion

The juli app modestly reduced average depressive symptoms in participants using the app for 8 weeks, with an increased probability of remission and MCID. However, attrition was high by the end of the trial and in our intention-to-treat analyses our primary endpoint was non-significant. As such, juli represents a low-risk addition to the care package of people with mild to severe depression. Further research is required to determine the most cost-effective technical support processes to enhance engagement and retention, and how juli could be implemented in current public health or clinical care models.

## Supplemental Material

sj-docx-1-dhj-10.1177_20552076241260409 - Supplemental material for Digitally managing depression: A fully remote randomised attention-placebo controlled trial Supplemental material, sj-docx-1-dhj-10.1177_20552076241260409 for Digitally managing depression: A fully remote randomised attention-placebo controlled trial by Aaron Kandola, Kyra Edwards, Marie AE Muller, Bettina Dührkoop, Bettina Hein, Joris Straatman and Joseph F Hayes in DIGITAL HEALTH

sj-doc-2-dhj-10.1177_20552076241260409 - Supplemental material for Digitally managing depression: A fully remote randomised attention-placebo controlled trial Supplemental material, sj-doc-2-dhj-10.1177_20552076241260409 for Digitally managing depression: A fully remote randomised attention-placebo controlled trial by Aaron Kandola, Kyra Edwards, Marie AE Muller, Bettina Dührkoop, Bettina Hein, Joris Straatman and Joseph F Hayes in DIGITAL HEALTH
